# The Care Pathway Delays of Cervical Cancer Patient in Morocco

**DOI:** 10.1155/2020/8796570

**Published:** 2020-08-18

**Authors:** Hind Mimouni, Khalid Hassouni, Boujemaa El Marnissi, Bouchra Haddou Rahou, Leila Alaoui, Rachid Ismaili, Abderraouf Hilali, Leila Loukili, Rachid Bekkali, Ahmed Nejmeddine

**Affiliations:** ^1^Hassan First University of Settat, Settat, Morocco; ^2^Radiotherapy Department, Hassan II University Hospital, Fez, Morocco; ^3^Research and Development Department, Hassan II University Hospital, Fez, Morocco; ^4^Research Department, High Institute of Nursing Professions and Technical Health, Rabat, Morocco; ^5^Cancer Research Institute, Fez, Morocco; ^6^Fondation Lalla Salma Prevention and Treatment of Cancers, Rabat, Morocco

## Abstract

**Introduction:**

The aim of this study is to document time intervals in cervical cancer care pathways, from symptom onset to disease detection and start of treatment, and evaluate how clinical, sociodemographic, and treatment factors influence delays throughout a patient's clinical pathway.

**Methods:**

A retrospective study was conducted at the FEZ Oncology Hospital of the Hassan II University Hospital Center in Morocco.

**Results:**

190 medical records of cervical cancer patients were collected. The dominant age group was 35–44, the median patient delay (PD) was 6 days, the median healthcare provider's delay (HCP) was 21 days, the median referral delay (RD) was 17 days, the median diagnostic delay (DD) was 9.5 days, the median total diagnostic delay (TDD) was 16 days, the median treatment delay (TD) was 67 days, and the median health system interval (HSI) was 92 days. Multivariate analysis revealed that age was associated with the patient delay, the healthcare provider's delay, the diagnosis delay, and the health system interval. The diagnosis year (the year in which the patient was diagnosed (either before 2012 or during 2012 as well as the other study years (from 2013 to 2017))), all investigations done prior to admission to the oncology hospital, and the age of first sexual activity were significantly associated with healthcare provider's delay.

**Conclusion:**

The integration of a model and standard care pathway into the Moroccan health system is essential in order to unify cervical cancer care in the country.

## 1. Introduction

Cervical cancer is the fourth most common cancer in women with approximately 570,000 new cases in 2018, representing 6.6% of all female cancers. About 90% of cervical cancer deaths occur in low- and middle-income countries. The high global cervical cancer mortality rate could be reduced through a comprehensive approach that includes effective prevention, early diagnosis, screening, and treatment programs. Virtually, all cervical cancers are associated with human papilloma virus (HPV) [1].

In Morocco, cervical cancer is the second most common cancer in women. 2258 new cases per year and 2465 annual deaths from cervical cancer were estimated in 2018 [2]. Incidence rates differ between the urban and rural areas. Most Moroccan women had never been screened for cervical cancer, and 70%–80% of all cervical cancer cases are diagnosed at an advanced stage [3].

Prolonged wait time in the delivery of cancer treatment is an important quality of care indicator, increasingly used to assess quality of oncology services, direct resource allocation, and program development [4]. It has become apparent that the measurement of care time intervals is complex and definition of the care interval monitored may bias the detection of change in wait times [5]. Organized cervical cancer screening was not available in Morocco until 2010 [3].

In 2010, a partnership was launched between the Moroccan Ministry of Health, the Lalla Salma Foundation for the Prevention and Treatment of Cancer, the United Nations Population Fund, and the screening group of the International Agency for Research on Cancer to implement a national programme for the early detection of cervical cancer by visual inspection using acetic acid (VIA). A pilot screening program was implemented during the period 2011–2013, which revealed a low compliance rate with screening; only 6% of women in the target age range (30–49 years) were screened for cervical cancer; the rate of treatment of precancerous lesions with LEEP (loop electrosurgical excisional excision procedure) was low (18%), but the baseline rate of VIA-positive women who underwent colposcopy was high (70%) [3].

Recently, the concept of delayed diagnosis has become as an important issue. It is categorized into four components including patient delay, healthcare provider's delay, referral delay, and system delay [6,7]. Therefore, this study aimed to document time intervals in cervical cancer care pathways, from symptoms onset to disease detection and start of treatment, between 2013 and 2017, and assess how clinical, sociodemographic, and treatment factors influence delays.

## 2. Materials and Methods

### 2.1. Study Design

A retrospective study was carried out at the FES hospital of oncology in Morocco.

### 2.2. Data Collection

**Figure 1:**
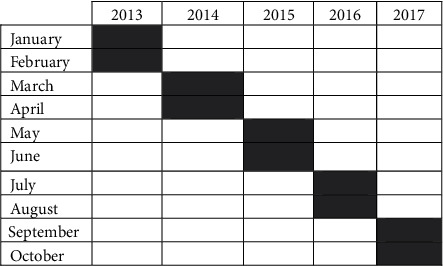
Distribution of medical records of cervical cancer patients for months and years 2013-2017 (5 years).

A form was used to extract information from the medical records of cervical cancer patients at the FEZ Oncology Hospital. This form was used to track the trajectory of patients through the health system during the periods of detection, investigation, and treatment. Patients included in the study were women registered with the diagnosis of invasive cervical cancer and excluded were those whose histopathology confirmed the absence of malignancy and that cancer was not the first primary cancer. 190 medical records of cervical cancer patients were collected. Medical charts of cervical cancer patients extracted for the months and years 2013–2017 (5 years) are given in Figure 1.

The survey includes sociodemographic and clinical characteristics and information about delays. The form was designed and developed in collaboration between the International Agency for Research on Cancer (IARC) and the Lalla Salma Foundation for Cancer Prevention and Control, whose validity was assessed by Moroccan medical experts in oncology.

### 2.3. Delays of Care Pathway

The care pathway concept is one of the concepts in the field of health management that addresses temporal and spatial issues and describes the analysis, design, planning, and control of all the steps required to provide service to a patient. It is categorized into the following components.

#### 2.3.1. Patient Delay

Patient delay is the period between the discovery of symptoms in the patient and the first medical consultation [7].

#### 2.3.2. Healthcare Provider's (HCP) Delay

Healthcare provider's (HCP) delay is the period between the patient's first presentation to the healthcare provider (HCP) and date of referral to the cancer diagnostic center [8].

#### 2.3.3. Referral Delay

Referral delay is the time interval between the date of final return of the patient at the diagnostic center with suspicion of cervical cancer and the date of the first appointment by the healthcare provider in the cervical cancer diagnostic center [8].

#### 2.3.4. Diagnostic Delay

Diagnostic delay is the time for all the investigations carried out at the diagnostic center [8].

#### 2.3.5. Total Diagnostic Delay

The total diagnostic delay is the period between onset of symptoms and confirmed diagnosis of cervical cancer [9].

#### 2.3.6. Treatment Delay

Treatment delay is the period between the confirmation of the diagnosis of cervical cancer and the start of treatment [10].

#### 2.3.7. Health System Interval

The health system interval is the time interval between the date of the first presentation to a healthcare provider (HCP) and the date of start of treatment [10].

### 2.4. Data Entry

Responses to the questionnaires were entered in a database specifically designed for this study. The whole study was monitored by the National Institute of Research on Cancer (IRC) in FEZ. The whole anonymized dataset is accessible by the IARC.

### 2.5. Statistical Analysis

We used simple descriptive analyses with frequency tables, calculation of mean durations, and univariate and multivariate analysis.

### 2.6. Ethical Approval

This study plan has been approved by the hospital-university of FEZ ethics committee.

## 3. Results

### 3.1. Characteristics of Cervical Cancer Patients

Table 1 shows the clinical and sociodemographic characteristics of the study participants. 190 medical records of cervical cancer patients were collected. 32% patients were classified in the 35–44 age group, followed by 23% with an age between 25 and 34 years. Two-thirds (74.21) of the patients were illiterate, and most of them (61.05%) were from urban areas. 60.35% of the participants are currently divorced, and 61.05% of patients were postmenopausal women. Almost the majority of patients (93.16) had a social coverage, and only 6.32% of patients had an active profession. Regarding the clinical details of study participants, 40% were classified as stage IIB (FIGO stage) and half of them (50.53) had a moderately differentiated tumor grade.

### 3.2. Care Pathway Delays

Table 2 presents the delays of care pathway for patients with cervical cancer, median patient delay (PD) of 6 days, median healthcare provider's delay (HCP) of 21 days, median referral delay (RD) of 17 days, median diagnostic delay (DD) of 9.5 days, median total diagnostic delay (TDD) of 16 days, median treatment delay (TD) of 67 days, and median health system interval (HSI) of 92 days.

### 3.3. Evolution of the Care Pathway over the Years

Table 3 presents the evolution of the care pathway over the years (2012–2017).The median patient waiting time before the year 2012 was 07 days and it was 04 days for the year 2017, which has been decreasing over the years. For the healthcare provider, the delay was 1811 days before 2012; this has been improved and decreased to 35 days in 2017. However, the referral delay has increased significantly over the years to 51 days in 2016 and 32 days in 2017 instead of 09 days in 2012. However, the total time to diagnosis has improved slightly and has been reduced from 39 days to 28 days. Concerning the treatment delay, it has an elevation between the years, 38 days before 2012, 123 days in 2012, 90 days in 2014, and 44 days in 2017. Similarly, the health system interval has increased between years, 72 days before 2012, 119 days in 2014, and 98 days in 2017.

### 3.4. Association of Cervical Cancer Delay and Sociodemographic and Clinical Characteristics

Table 4 shows the association between cervical cancer delay and sociodemographic and clinical characteristics. Univariate analysis found that variables such as diagnosis year was significantly associated with PD (*P*=0.01), HCP (*P* ≤ 0.001), RD (*P* ≤ 0.001), TDD (*P*=0.04), and TD (*P* ≤ 0.001).Other variables such as educational level was associated with RD (*P*=0.03), DD (*P*=0.01), and HSI (*P* ≤ 0.001).

### 3.5. Multivariate Linear Regression Analysis of Sociodemographic and Clinical Characteristics with Cervical Cancer Delay

Table 5 shows the multivariate linear regression analysis of sociodemographic and clinical characteristics with cervical cancer delay. Regarding the sociodemographic characteristics, the age category 45–54 was chosen as the reference modality for all delays. The patient delay was significantly associated with the age category of 35–44 years (*P*=0.042); the patient delay was 2.1 times more in women between 35 and 44 years than in those between 45 and 54 years.

Concerning the healthcare provider's delay, a significant correlation has been shown with women over 65 years of age (*P*=0.025); these patients are once more delayed than women in the age category between 45 and 54 years of age. Thus, the time to diagnosis was 3.14 times longer in patients in the 35–44 age group than in those in the 45–54 age group (*P* ≤ 0.001). However, a significant association was found between health system interval time and marital status, with the interval time being 0.385 longer for widowed women than married women. Yet, the age of first sexual activity for women was associated with healthcare provider's delay (*P*=0.015), diagnosis delay (*P*=0.013), and treatment delay (*P*=0.031).

The health system interval was 0.05 less in patients referred by a specialist physician compared to those referred by a private physician. For tumor grade, it was associated with the treatment delay (*P*=0.042), and the duration was almost 30 times longer in patients with a poorly differentiated grade compared to those with a well-differentiated grade.

For patients who completed the investigation prior to the oncology center, the healthcare provider's delay was 1.02 times longer and the referral delay was 1.27 less than those who did not complete the investigation prior to care at the oncology center.

## 4. Discussion

This study identified different delays in the care pathway of cervical cancer. The total interval was categorized into patient delay, healthcare provider's delay, referral delay, diagnosis delay, and treatment delay. Results revealed that the median patient delay was 6 days, median healthcare provider's delay was 21 days, median referral delay was 17 days, median diagnostic time was 9.5 days, median total diagnosis delay was 16 days, median treatment delay was 67 days, and median health system interval was 92 days. The total diagnosis delay of more than 90 days was defined as “long diagnostic delay” and 90 days or less as “short diagnostic delay” [9]. The current study showed that the median total diagnosis was 16 days, which means a short diagnostic delay; this is merit to the efforts made at the strategic level and also at the oncology hospital; the introduction of awareness and screening campaigns, as well as equipping regional hospitals with screening facilities, has played a crucial role in reducing the time to diagnosis. This is consistent with another study that assert the median total diagnostic delay as 68 days, and 48 (39.3%) patients had delayed diagnosis of cervical cancer [11]. However, the study conducted in Nepal showed that the median total diagnostic delay was 157 days with more than three-fourths (77.3%) of the patients having longer a total diagnostic delay of >90 days [6].

Out of the total diagnostic delay, median patient delay, median healthcare provider's delay, median referral delay, and median diagnostic waiting time were 68.5 days, 40 days, 5 days, and 9 days, respectively [8]. Majority of the patients had experienced longer delay of each type except referral delay, 57% of patients had experienced longer patient delay of >60 days, 90% had suffered longer healthcare provider's delay of >1 week, 31.8% had longer referral delay of >1 week, and 66.2% had waited >1 week at the diagnostic center for final diagnosis [8], which is at variance with the literature which reveals that the healthcare provider (HCP) delay is defined as the time period between the patient's first presentation to healthcare provider (HCP) and final referral by HCP at the cancer diagnostic center. The period of seven days or less has been defined as a “short HCP period” and more than seven days has been described as “long HCP delay” [8]. In this study, the healthcare provider's delay is therefore considered a long delay (21 days).The period of seven days or less has been defined as “short RD” and more than seven days has been referred as “long RD” [8], which signifies that the referral delay (17 days) is considered in the present study as a long delay.

Variation in each type of delay was observed among women with different attributes and in context of healthcare service delivery [11].

In line with other research on the care pathway, especially the patient delay and the diagnosis delay of cervical cancer, the findings show that the median time from the symptom triggering presentation to presentation was one month and the median time from presentation to diagnosis was three months [12]. This is at variance with the present study, which found that the median patient delay was 6 days and the median diagnostic time was 9.5 days. Furthermore, the duration of more than 60 days was defined as long patient delay and 60 days or less was defined as short patient delay [11]. Diagnostic waiting time includes waiting time for all relevant investigations of symptoms in the diagnostic center. The period of seven days or less was defined as short waiting time and more than seven days was defined long waiting time [8]. Among all the components, patient delay was the major contributor to the diagnostic delay [7].

Concerning the median treatment delay, it was 67 days in our study. Similar studies have been developed in terms of treatment delay that showed that the patients generally sought treatment within 90 days after diagnosis. Although, intervals of >180 days between diagnosis and treatment were associated with a higher likelihood of death than are intervals of <90 days [13].

Multivariable analysis revealed that the age was associated with patient delay (*P*=0.042), the healthcare provider (*P*=0.025), diagnosis delay (*P* < 0.001), and health system interval (*P*=0.018) and there is no relationship between the delay and the study level and the marital status. A study conducted in Sudan has shown that older age is a predictor for patient delay [14].

In line with other research on the cervical cancer delay, the findings show that the lower education status of husband, patient's positive history of sexually transmitted disease, nonattendance of screening program, and not performing cervical/per speculum examination during initial consultation were significantly associated with longer diagnostic delays [7]. Almost all studies in our field of investigation have indicated that women's literacy is considered an independent risk factor for late diagnosis of cervical cancer. However, educational attainment is indirectly related to average income, knowledge, and understanding the nature of the disease, related risk factors, and health education [15,16].

The findings showed that the age of first sexual activity was associated with healthcare provider (*P*=0.015), diagnosis delay (*P*=0.013), and treatment delay (*P*=0.031). Furthermore, this study revealed that women with undifferentiated tumor grade were likely to have healthcare provider's delay and treatment delay. There was a significant association between the healthcare provider's delay and the diagnosis year (*P* ≤ 0.001) and all investigations done prior to admission to the oncology hospital (*P*=0.024). While our findings are consistent with some studies, they were in contrast with others. A study conducted in Nepal establishes the fact that healthcare provider's delay as another major delay in cervical cancer diagnosis [8]. Although in low proportion, longer medical delay has also been observed in previous studies, even in developed countries [7,17].

## 5. Conclusion

This study made it possible to diagnose the evolution of the delays over a period of five years in patients with cervical cancer. The results showed short delays and other long delays that need to be improved. The patient delay and the diagnosis delay were the short delays and the healthcare provider's delay, the referral delay, and the median diagnostic time were the long delays. However, the main factors associated with delays were the age, the age of first sexual activity, the tumor grade, the diagnosis year, and the period of investigation.

This research would make it possible to improve care pathways and, above all, long waiting times and to integrate a standard model of care pathway in all hospitals in Morocco.

## Figures and Tables

**Table 1:** Sociodemographic and clinical characteristics of participants.

Variables	Number	Percent
Age		
<25	5	2.63
25–54	144	75.79
55–64	34	17.89
Not available	7	3.68
Residence		
Rural	72	37.89
Urban	116	61.05
Not available	2	1.05
Social coverage		
Yes	177	93.16
No	11	5.79
Not available	2	1.06
Educational level		
None	141	74.21
Primary school	17	8.95
Secondary school	16	8.42
Superior	06	3.16
Not available	10	5.26
Marital status		
Single	5	2.63
Married	15	7.89
Divorced	115	60.53
Widow	35	18.42
Not available	20	10.53
Active profession		
Yes	12	6.32
No	172	90.53
Not available	3.16	
Menopause		
Yes	116	61.05
No	61	32.11
Not available	13	6.84
FIGO stage		
Not available	8	4.21
Stage IA	9	4.74
Stage IB	24	12.63
Stage IIA	25	13.16
Stage IIB	76	40.00
Stage IIIA	12	6.32
Stage IIIB	16	8.42
Stage IVA	5	2.63
Stage IVB	9	4.74
Unknown	6	3.16
Tumor grade		
Well differentiated	43	22.63
Little differentiated	25	13.16
Moderately differentiated	96	50.53
Others	3	1.58
Not available	23	12.1

**Table 2:** Care pathway delays.

Variables	*N*	Mean	SD	Median (min–max)
Patient delay	172	6.8	4.3	6 (1–28)
Healthcare provider's delay	160	237	689	21 (0–3562)
Referral delay	170	61	139	17 (0–1517)
Diagnosis delay	170	69	385	9.5 (0–3665)
Total diagnosis delay	166	78	389	16 (2–3672)
Treatment delay	151	82	61	67 (0–401)
Health system interval	160	106	70	92 (5–401)

**Table 3:** Evolution of the care pathway over the years.

Diagnosis year	Median						
	PD	HaCP	RD	DD	TDD	TD	HSI
≤2012	7	1811	9	1820	39	38	72
2012	5	21	33	72	7.5	123	114
2013	6	15	9	30	13	84	108
2014	7	15	37	66	18	90	119
2015	6	21	21	56	18	59	81
2016	6.5	10	51	76	14	54	70
2017	4	35	32	71	28	44	98

PD: patient delay; HCP: healthcare provider; RD: referral delay; DD: diagnosis delay; TDD: total diagnosis delay; TD: treatment delay; HSI: health system interval.

**Table 4:** Association of cervical cancer delay and sociodemographic and clinical characteristics.

Variables	PD	HCP	RD	DD	TDD	TD	HSI
Age	0.31	0.30	0.94	0.73	0.53	0.78	0.61
Marital status	0.81	0.58	0.12	0.20	0.24	0.81	0.20
Educational level	0.44	0.60	0.03	0.01	0.50	0.32	0.00
Age of first sexual activity	0.00	0.00	—	0.63	0.23	0.27	0.00
Investigation before the cancer center	0.88	0.01	0.00	0.66	0.15	0.02	0.02
Nature of consultation	0.40	0.55	0.01	0.20	0.00	—	0.00
Hospital referrer	0.34	0.00	0.00	0.65	0.09	—	0.67
Diagnosis year	0.01	0.00	0.00	0.34	0.04	0.00	0.08
Multistakeholder consultation meeting	—	—	—	—	—	0.00	0.00

PD: patient delay; HCP: healthcare provider; RD: referral delay; DD: diagnosis delay; TDD: total diagnosis delay; TD: treatment delay; HSI: health system interval.

**Table 5:** Results of multiple linear regression between cervical cancer delay and sociodemographic and clinical factors (*n* = 190).

Variables	Patient delay (*β* (95% CI) *P* value)	Healthcare provider (*β* (95% CI) *P* value)	Referral delay (*β* (95% CI) *P* value)	Diagnosis delay (*β* (95% CI) *P* value)
Age				
25–34	1.64 (−2.4; 5.67) 0.423	−1.18 (−3.13; 0.761) 0.229	−0.7 2 (−3.45; 2.01) 0.601	2.57 (−0.643; 5.78) 0.113
35–44	2.1 (0.0748; 4.13) 0.042	−0.151 (−1.06; 0.76) 0.743	−0.714 (−1.51; 0.789) 0.077	3.14 (1.35; 4.93) 0.001
45–54 (ref)				
55–64	0.542 (−1.26; 2.34) 0.553	0.233 (−0.42; 0.886) 0.48	0.335 (−0.35; 1.02) 0.334	−0.0891 (−1.21; 1.03) 0.872
>65	0.65 (−1.23; 2.53) 0.495	0.951 (0.121; 1.78) 0.025	−0.16 (−0.958; 0.638) 0.691	−0.386 (−1.64; 0.865) 0.534
Marital status				
Divorced	—	−0.438 (−1.23; 0.357) 0.276	−0.582 (−1.47; 0.309) 0.198	—
Widow		−0.317 (−1.08; 0.45) 0.413	−0.238 (−0.939; 0.464) 0.502	
Married (ref)				
Study level				
Primary school		0.181 (−0.979; 1.02) 0.971	0.507 (−0.401; 1.41) 0.270	—
Secondary school	—	0.229 (−0.571; 1.03) 0.571	−0.238 (−0.939; 0.464) 0.502	
None (ref)				
Age of first sexual activity	0.0381 (−0.175; 0.251) 0.724	0.896 (0.178; 1.61) 0.015	−0.032 (−0.135; 0.0712) 0.539	−0.238 (−0.423;−0.0531) 0.013
Hospital referrer				
Self-reference	−0.531 (−3.1; 2.04) 0.683	0.428 (−0.681; 1.54) 0.445	0.156 (−0.9090; 1.22) 0.771	0.0987 (−1.42; 1.62) 0.895
General practitioner	1.51 (−1.17; 4.2) 0.266	−0.469 (−2.04; 1.1) 0.553	0.162 (−1.01; 1.33) 0.783	−0.476 (−1.81; 0.858) 0.473
Specialist physician	−0.923 (−2.4; 0.554) 0.219	0.875 (0.238; 1.51) 0.008	−0.342 (−1.02; 0.335) 0.318	−0.0239 (−1.04; 0.994) 0.962
Private physician (ref)				
Stage				
I	−1.2 (−2.99; 059) 0.187	1.98 (−0.445; 0.84) 0.542	0.457 (−0.21; 1.12) 0.177	0.11 (−0.951; 1.17) 0.834
II (ref)				
III	−0.862 (−0.273; 1.01) 0.364	−0.264 (−0.973; 0.446) 0.462	−0.197 (−0.96; 0566) 0.609	−0.917 (−2.67; 0.837) 0.295
IV	−1.56 (−4.04; 0.923) 0.216	0.54 (−0.418; 1.5) 0.265	−0.52 (−1.44; 0.397) 0.263	−0.698 (−1.87; 0.478) 0.236
Tumor grade				
Well-differentiated (ref)				
Undifferentiated	—	0.848 (0.749; 1.62) 0.032	0.699 (−0.118; 1.52) 0.093	0.163 (−0.776; 1.1) 0.727
Mildly differentiated	—	0.683 (−0.517; 0.654) 0.817	0.21 (−0.374; 0.794) 0.477	−0.702 (−1.65; 0.241) 0.139
Diagnosis year				
<2012 (ref)				
2012	3.05 (0.16; 5.94) 0.039	−3 (−4.02;−1.97) 0.001	0.754 (−0.323; 1.83) 0.167	0.169 (−1.54; 1.88) 0.842
2013	0.648 (−1.72; 3.01) 0.588	−3.5 (−4.38;−2.62) 0.001	−0.321 (−1.27; 0.632) 0.505	−0.106 (−1.89; 1.68) 0.905
2014	3.04 (0.684; 5.39) 0.012	−3.18 (−4.19;−2.16) 0.001	0.557 (−0.527; 1.64) 0.309	0.953 (−0.705; 2.61) 0.250
2015	2.06 (−0.38; 4.51) 0.097	−2.98 (−3.89;−2.06) 0.001	−0.29 (−1.28; 0.702) 0.562	−0.457 (−2.2; 1.29) 0.597
2016	2.8 (0.568; 5.03) 0.014	−3.42 (−4.56;−2.27) 0.001	0.48 1 (−0.586; 1.55) 0.372	1.11 (−0.771; 2.99) 0.238
2017	2.01 (−0.395; 4.41) 0.101	−2.87 (−3.9;−1.85) 0.001	0.488 (−0.584; 1.56) 0.368	—
Investigation before the oncology center				
Yes	—	1.02 (0.136; 0.191) 0.024	−1.27 (−1.95; −0.583) 0.001	001–0.569 (−1.45; 0.309) 0.196
No (ref)				
Variables	Total diagnosis delay (*β* (95% CI) *P* value)	Treatment delay (*β* (95% CI) *P* value)	Health system interval (*β* (95% CI) *P* value)	
Age				
25–34	−0.406 (−2.22; 1.41) 0.658	4.64 (−70.6; 79.9) 0.903	0.0465 (−1.03; 1.13) 0.932	
35–44	0.409 (−0.41; 1.23) 0.324	11.1 (−15.3; 37.5) 0.405	0.0612 (−0.355; 0.477) 0.770	
45–54 (ref)				
55–64	0.196 (−0.381; 0.772) 0.502	2.32 (−23.1; 27.7) 0.856	0.242 (−0.121; 0.605) 0.188	
>65	0.237 (−0.424; 0.898) 0.478	−25.3 (−53.4; 2.81) 0.077	−0.493 (−0.899; −0.874) 0.018	
Marital status				
Divorced	−0.322 (−1.08; 0.436) 0.401	−12.4 (−45.8; 20.9) 0.460	−0.488 (−0.952; −0.025) 0.039	
Widow	0.131 (−0.471; 0.734) 0.667	18.7 (−7.84; 45.2) 0.165	0.385 (0.0176; 0.753) 0.040	
Married (ref)				
Study level				
Primary school	−0.0135 (−0.689; 0.662) 0.969	0.207 (−32.3; 32.7) 0.990	0.165 (−0.26; 0.59) 0.442	
Secondary school	0.139 (−0.621; 0.898) 0.718	−17.5 (−52.6; 17.5) 0.322	−0.377 (−0.838; 0.0845) 0.108	
None (ref)				
Age of first sexual activity	—	−3.55 (−6.78; −0.323) 0.031	0.038 (−0.00491; 0.0808) 0.082	
Hospital referrer				
Self-reference		—	−0.163 (−0.706; 0.381) 0.0.554	
General practitioner	—		−0.829 (−1.43;−0.225) 0.008	
Specialist physician			−0.049 (−0.394; 0.296) 0.778	
Private physician (ref)				
Stage				
I	−0.3 (−0.864; 0.264) 0.294	−20.7 (−45.2; 3.8) 0.097	−0.233 (−0.6; 0.133) 0.209	
II (ref)				
III	−0.431 (−1.02; 0.157) 0.149	−71 (−33.8; 19.6) 0.599	−0.167 (−0.55; 0.216) 0.388	
IV	−0.619 (−1.4; 0.158) 0.117	−0.585 (−36.4; 35.2) 0.974	−0.0316 (−0.528; 0.465) 0.90	
Tumor grade				
Well-differentiated (ref)				
Undifferentiated	0369 (−0.286; 1.02) 0.266	29.8 (1.08; 58.5) 0.042	0.373 (−0.0449; 0.79) 0.080	
Mildly differentiated	−0.333 (−0.826; 0.161) 0.184	−6.53 (−29; 16) 0.565	−0.0447 (−0.363; 0.274) 0.781	
Diagnosis year				
<2012 (ref)				
2012	−1.2 (−2.1;−0.307) 0.009	−26.5 (−70.9; 17.8) 0.238	0.026 (−0.599; 0.651) 0.943	
2013	−0.956 (−1.79; −0.118) 0.026	−23 (−58.9; 12.9) 0.206	−0.0659 (−0.614; 0.482) 0.812	
2014	−0.66 (−1.57; 0.248) 0.152	−21.7 (−58.1; 14.6) 0.238	0.261 (−0.349; 0.871) 0.397	
2015	−0.562 (−1.37; 0.245) 0.170	−43.9 (−76.9; −10.9) 0.010	−0.00165 (−0.555; 0.552) 0.995	
2016	−0.51 (−1.39; 0.365) 0.250	−28.3 (−67.2; 10.6) 0. 151	0.163 (−0.43; 0.757) 0.585	
2017	−0.209 (−1.06; 0.64) 0.626	−30.8 (−68.4; 6.84) 0.108	−0.0885 (−0.663; 0.486) 0.760	
Investigation before the oncology center				
Yes	−0.359 (−0.922; 0.203) 0.208	−5.54 (−30.3; 19.3) 0.658	—	
No (ref)				

Significant at *P* value < 0.05/model included variables that were significant on univariate analyses at the *P* (0.05 level).

## Data Availability

The data used to support the conclusions of this study are included in the article.

## Abbreviations

PD: Patient delay
HCP: Healthcare provider
RD: Referral delay
DD: Diagnosis delay
TDD: Total diagnosis delay
TD: Treatment delay
HSI: Health system interval.

## Additional Points

The main limitation of this study is the lack of some data during the research since it is a retrospective study and the data collection was done through patient medical records.
